# Effectiveness of interventions based on patient empowerment in the control of type 2 diabetes in sub‐Saharan Africa: A review of randomized controlled trials

**DOI:** 10.1002/edm2.174

**Published:** 2020-08-25

**Authors:** Amélie Mogueo, Charity Oga‐Omenka, Marie Hatem, Barthelemy Kuate Defo

**Affiliations:** ^1^ School of Public Health of the University of Montreal Montreal QC Canada; ^2^ Public Health Research Institute of the University of Montreal Montreal QC Canada; ^3^ Sainte‐Justine University Hospital Centre Montreal QC Canada

**Keywords:** effectiveness, intervention, patient empowerment, self‐management, sub‐Saharan Africa

## Abstract

**Background:**

It is estimated that 1.6 million deaths worldwide were directly caused by diabetes in 2016, and the burden of diabetes has been increasing rapidly in low‐ and middle‐income countries. This study reviews existing interventions based on patient empowerment and their effectiveness in controlling diabetes in sub‐Saharan Africa.

**Method:**

PubMed, MEDLINE, EMBASE, CINAHL, Web of Science, PsycINFO and Global Health were searched through August 2018, for randomized controlled trials of educational interventions on adherence to the medication plan and lifestyle changes among adults aged 18 years and over with type 2 diabetes. Random‐effects meta‐analysis was used.

**Results:**

Eleven publications from nine studies involving 2743 participants met the inclusion criteria. The duration of interventions with group education and individual education ranged from 3 to 12 months. For six studies comprising 1549 participants with meta‐analysable data on glycaemic control (HbA1c), there were statistically significant differences between intervention and control groups: mean difference was −0.57 [95% confidence interval (CI) −0.75, −0.40] (*P* < .00001, *I* ^2^ = 27%). Seven studies with meta‐analysable data on blood pressure showed statistically significant differences between groups in favour of interventions. Subgroup analyses on glycaemic control showed that long‐term interventions were more effective than short‐term interventions and lifestyle interventions were more effective than diabetes self‐management education.

**Conclusion:**

This review supports the findings that interventions based on patient empowerment may improve glycaemia (HbA1c) and blood pressure in patients with diabetes. The long‐term and lifestyle interventions appear to be the most effective interventions for glycaemic control.

## Graphical abstract

This original research is, to our knowledge, the first systematic review done in context sub‐Saharan Africa showing the effectiveness of existing interventions based on patient empowerment in the control of type 2 diabetes. Our study found that interventions based on patient empowerment may improve glycaemia (HbA1c) and blood pressure (BP) in patients with type 2 diabetes. It also found that the long‐term and lifestyle interventions appear to be the most effective interventions for glycaemic control.

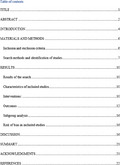

## 1 INTRODUCTION

Patient empowerment has evolved since the Alma‐Ata conference in 1978 into one of the health promotion strategies in the Ottawa Charter of 1986, and nowadays as one of the general principles of the World Health Organization (WHO)'s Global action plan for the prevention and control of noncommunicable diseases (NCDs) 2013‐2020.^1^ Chronic NCDs such as diabetes are among the leading causes of morbidity and mortality in sub‐Saharan Africa (SSA).^2^, ^3^ Diabetes is a long‐term management disease, and its management is quite expensive for patients and their families who carry its financial burden in SSA, given the shortfall or nonexistence of the health insurance system.^4^, ^5^, ^6^ It is characterized in much of SSA by a preponderance of patients' nonadherence to therapeutic plans, and there is an urgent need to implement cost‐effective patient‐based interventions that empower patients to control their own disease.

Self‐management of diseases^7^ or patient empowerment^8^ is broadly defined as the most important decision taken by the patient affecting the diabetic patient's health and well‐being. Based on such definition, the content, complexity and effectiveness of patient empowerment interventions vary significantly from one study to another. It varies in terms of study's aims, target behaviours (eg self‐monitoring of blood glucose, diet or exercise), intensity, duration, place of delivery (eg clinic‐ or community‐based), mode of delivery (eg group or individual), type and training of the facilitator (eg physician, nutritionist, nurse or peer) and theoretical underpinnings.^8^, ^9^ Indeed, the development of interventions based on the patient empowerment approach has been influenced by several theories of health behaviour change.^10^, ^11^, ^12^ Antonovsky^13^ proposed the salutogenic theory to summarize and operationalize patient empowerment in three dimensions: intelligibility, manageability and meaningfulness from the patient's perspective. These three dimensions constitute the sense of coherence (SOC), and a stronger SOC is predictive of salutogenesis or a production of health. He also recommended the presence of internal and external resources as prerequisites to develop a stronger SOC for patient empowerment.^14^, ^15^ Therefore, interventions that integrate the three dimensions of SOC and the resources for patients are more likely to be effective for disease self‐management by the patients.^14^

Increasingly, scientific evidence supports the hypothesis that patient empowerment interventions improve patients' abilities, allowing them to better control their biochemical and physical parameters as well as their lifestyle.^9^, ^16^, ^17^ Several systematic reviews have been conducted in high‐income countries, sometimes showing inconsistent effects of patient empowerment in the control of chronic NCDs.^18^, ^19^ In a study on an African American population bearing a disproportionate burden of diabetes and its complications, Ricci‐Cabello et al (2013) showed that PE interventions could be at least partially effective in improving both processes of care and health outcomes. To our knowledge, no review has been conducted so far in SSA. Positive effects recorded in some interventions^20^, ^21^ have been highly variable from one intervention to another,^22^, ^23^, ^24^ and even not statistically significant in others.^25^, ^26^, ^27^ This variability of interventions based on patient empowerment makes it difficult to assess their effectiveness, thereby limiting their usefulness in the decision‐making process for the improvement of the quality of health care without a measurement of their effect sizes. This review considers the following research question: What are the existing interventions based on patient empowerment and their effectiveness in controlling diabetes in SSA?

## 2 MATERIALS AND METHODS

The review was registered in PROSPERO (registration number: CRD42018095070).

### 2.1 Inclusion and exclusion criteria

#### 2.1.1 Participants

Only studies conducted in sub‐Saharan Africa among adult patients aged 18 and over with type 2 diabetes mellitus^28^ were selected. There were no restrictions on patient sociodemographic characteristics, the background of the person providing the patient educational empowerment, the sample size or the target groups. Studies carried out on mixed populations of patients with type 1 and type 2 diabetes were excluded from this review because the results were not reported separately for type 1 and type 2 diabetes; our focus being on type 2 diabetes, it was not possible to extract relevant data.

#### 2.1.2 Interventions

All selected studies were randomized controlled trials of an educational intervention: diabetes self‐management education, pharmacist‐led intervention, lifestyle education programmes, and cognitive behavioural coaching and peer‐led intervention. These interventions aimed to lead the patient to be able to self‐manage type 2 diabetes (T2D) in terms of adherence to the medication plan, lifestyle changing and follow‐up. The interventions varied in duration, intensity, frequency, strategy, topics and educational content. The self‐management of T2D was analysed using the three dimensions of the salutogenic theory: intelligibility (knowledge about T2D and related factors, disease process, complications and treatment options), manageability (taking medication, self‐monitoring of blood glucose, insulin titration, measurement of food intake, frequent exercise and follow‐up) and meaningfulness (psychosocial support).^17^ Only studies describing interventions and the process of empowering T2D patients were included (Appendix S1).

#### 2.1.3 Control

The control or comparison group was the treatment as usual/standard care.

#### 2.1.4 Outcomes

##### Primary outcomes

The two primary outcomes were the glycosylated haemoglobin (HbA1c) or fasting blood sugar (FBS) and self‐efficacy in disease control. They are the primary outcomes used in the literature as direct outcomes when evaluating the effectiveness of intervention based on patient empowerment targeting diabetic patients.^19^, ^29^

##### Secondary outcomes

The secondary outcomes were blood pressure (BP), lipid profile parameters (total cholesterol), physical parameters (body mass index) and lifestyle (diet, physical activity, smoking and alcoholism). When available, the use of services (hospital admission) and medication adherence were evaluated as secondary outcomes. Secondary outcomes of interest were outcomes often used in the literature as indirect outcomes to evaluate the effectiveness of intervention based on patient empowerment targeting diabetic patients.^29^

### 2.2 Search methods and identification of studies

#### 2.2.1 Electronic searches

A systematic review was conducted of published studies until 31 July 2018, using the Preferred Reporting Items for Systematic Review and Meta‐Analysis (PRISMA).^30^ The search strategy included only terms (and synonyms) relating to or describing interventions focused on patient empowerment in the management of diabetes. Seven databases were used: PubMed, MEDLINE, EMBASE, CINAHL, Web of Science, PsycINFO and Global Health. Google, ProQuest Dissertations, Global Theses and GraySource Index were explored for grey literature. Only French and English studies were included in this review. The University of Montreal Paramedical Librarian cross‐checked the research's strategy. Before the final analysis, we checked the alert system in each database to ensure that all the new relevant studies were retrieved for inclusion in this review.

#### 2.2.2 Data extraction

**FIGURE 1:**
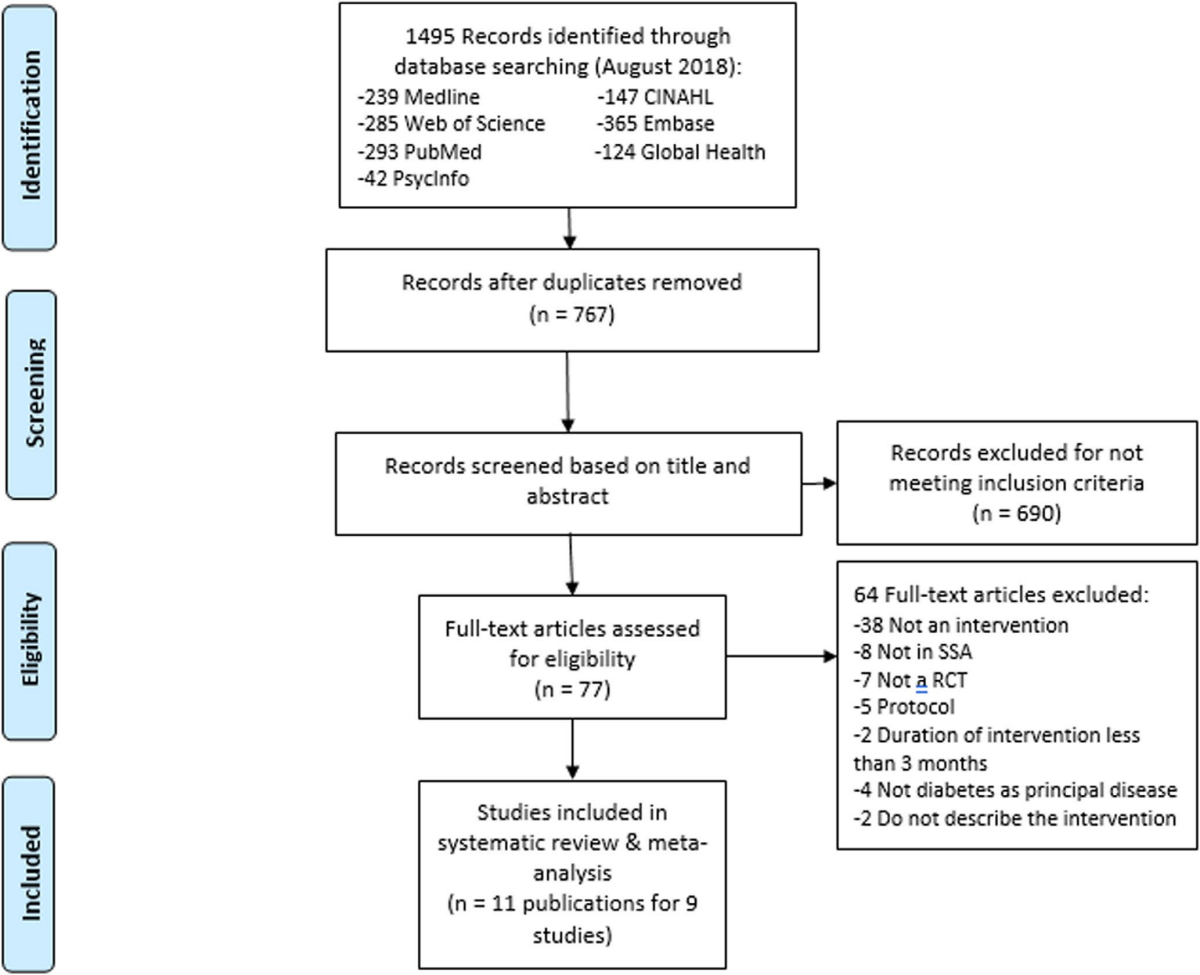
PRISMA flow chart of the selection process

Two authors (AM and CO) independently reviewed all studies based on inclusion criteria, starting with the title and abstracts, and through the full publications to generate a final selection. In cases of disagreement between the two authors on the eligibility of a study, a discussion with the senior author (BKD) was necessary to find a point of agreement. An adapted PRISMA flow chart of study selection was used (Figure 1).^31^ The two review authors independently extracted the data from studies that met the inclusion criteria using a summary table (Table 1). Any disagreements were resolved by discussion, and if required by the senior author. The original authors of each publication were contacted for any relevant missing information on the trial.

**TABLE 1:** Characteristics of included studies

Authors, publication year	Country (region of residence), type of place of residence	Population size (N) and number of women (n)	Mean age (SD)	Type of participants	Description of intervention	Mode of delivery (number of participants)	Duration of the intervention (mo)	Lost to follow‐up at the end of the intervention	Type of the facilitator	Main outcomes: (1) primary outcomes and (2) secondary outcomes
Amendezo et al, 2017^24^	Rwanda (Kigali), Urban	N = 223; n = 166	51.50 (11)	T2D	Intervention group: 115 patients: Lifestyle intervention and usual care. Frequency: Monthly lifestyle group counselling and education sessions over 12 mo. Intensity: 45‐60 min. Topics: Diabetic diet, regular physical activity, cessation of smoking, and alcohol abuse, adherence to medications and to regular medical follow‐up, diabetic complications screening and treatment, self‐management of hypoglycaemia and hyperglycaemia and stress management. Supports: Educational pamphlets Framework or theoretical approach: Patient‐centred approach Control group: 108 patients: Usual care Frequency: Monthly medical follow‐up and individual counselling on dietary habits and lifestyle change over 12 mo. Intensity: Not specified	Group education (not specified)	12	28	Physician, nurse, dietician, psychologist	(1) Significant difference between groups in HbA1c (*P* < .001). (2) Significant difference between groups in SBP (*P* = .005), DBP (*P* = .02), weight (<0.001)
Debussche et al, 2018^39^	Mali (Bamako), Urban	N = 151; n = 115	52.50 (9.80)	T2D	Intervention group: 76 patients: Peer‐led structured patient education and usual care. Frequency: One course every 3 mo over 12 mo. Intensity: 1.5‐2 h Topics: Cardiovascular risk management, food intake, exercise, blood glucose and insulin management. Supports: Specific booklets Framework or theoretical approach: Empowerment‐based approach Control group: 75 patients: Usual care. Frequency: One visits every 3 mo for regular follow‐ups Intensity: Not specified	Group education (4‐10 participants)	12	IG: 6, CG: 5	Peer	(1) Significant difference between groups in HbA1c (*P* = .006). No significant difference between groups in diabetes knowledge score (*P* = .17). (2) Significant difference between groups in SBP (*P* = .003) and BMI (*P* = .0005). No significant difference between groups in DBP (*P* = .36)
Erku et al, 2017^40^	Ethiopia (Gondar), Urban	N = 127; n = 46	60.55 (12.45)	T2D	Intervention group: 62 patients: Pharmacist‐led medication therapy management and usual care. Frequency: One intensive education every 3 mo over 6 mo. Intensity: 45 min Topics: Patient's medication regimen, the role of balanced diet, regular exercise, smoking cessation. Supports: Charge‐free telephone counselling. Framework or theoretical approach: Personalized approach and tailored to the specific needs of each patient (patient‐centred approach). Control group: 65 patients: Usual care. Frequency: One short discussion with physician, every 3 mo over 6 mo. Intensity: 3‐4 min	Individual education	6	IG: 8, CG: 12	Pharmacist, physician	(2) Significant difference between groups in medication adherence (*P* < .01) and hospital admissions (*P* < .001)
Gathu et al, 2018^41^	Kenya (Nairobi), Urban	N = 140; n = 62	48.80 (9.80)	T2D	Intervention group: 55 patients: DSME and usual care Frequency: One session every 6 wk Intensity: 1 h. Topics: Being active, nutrition, monitoring blood glucose and adherence to medication. Supports: Diabetes booklet and graphic material illustrating several self‐care activities. Framework or theoretical approach: Patient‐centred approach. Control group: 41 patients: Usual care Frequency: Standard doctors' consultation in a quarterly basis (opportunity to learn about self‐management in a flexible and informal way). Intensity: 20‐30 min	Individual education	6	(IG) 15, (CG) 29	Physician	(1) No significant difference between groups in HbA1c (*P* = .37). (2) No significant difference between groups in SBP (*P* = .57), DBP (*P* = .39) and BMI (*P* = .86)
Hailu et al, 2018^42^	Ethiopia (Jimma), Urban and Rural	N = 220; n = 72	47 (10)	T2D	Intervention group: 116 patients: DSME and usual care. Frequency: One session every month for 6 consecutive months. Intensity: 1.5 h. Topics: Diabetes management, healthy foods, healthy physical exercise, food care practice, medication management, hypoglycaemia management, stress and depression self‐management. Supports: Handbooks and fliers with colourful, illustrative pictures customized to the local context and patients' literacy level and phone reminders, free charge for FBS test. Framework or theoretical approach: Diabetes self‐management approach. Control group: 104 patients: Usual care. Frequency: Six visits every month for 6 consecutive months. Intensity: Not specified	Group education (8‐12 participants)	6	IG: 38, CG: 40	Nurse	(1) No significant difference between groups in HbA1c (*P* = .20). (2) Significant difference between groups in SBP (*P* = .000) and DBP (*P* = .000)
Mash et al, 2012^35^	South Africa (Western Cape), Urban	N = 1570; n = 1158	56.10 (11.55)	T2D	Intervention group: 710 patients: DSME and usual care. Frequency: One session every 3 mo Intensity: 60 min Topics: Understanding diabetes, living a healthy lifestyle, understanding the medication and avoiding complications. Supports: Graphic materials, flipchart and various card games, bulk text message. Framework or theoretical approach: Motivational interviewing. Control group: 860 patients: Usual care. Frequency: Four routine visits. Intensity: Not specified	Group education (10‐15 participants)	12	IG: 385, CG: 319	Health promoter, physician, nurse	(1) No significant difference between groups in HbA1c (*P* = .967) and psychological factors (*P* = .52). (2) Significant difference between groups in SBP (*P* = .04) and DBP (*P* = .002). No significant difference between groups in total cholesterol (*P* = .066), weight (*P* = .392), medication adherence (*P* = .89), physical activity (*P* = .57), diet (*P* = .80), smoking (*P* = .8), quality‐of‐life measurements (*P* = .71) and ICER (cost‐effectiveness) = 1862$/QALY gained
Mash et al, 2015^36^	South Africa (Western Cape), Urban	N = 1570; n = 1158	56.10 (11.55)	T2D	Intervention group: 710 patients: DSME and usual care. Frequency: One session every 3 mo Intensity: 60 min Topics: Understanding diabetes, living a healthy lifestyle, understanding the medication and avoiding complications. Supports: Graphic materials, flipchart and various card games, bulk text message. Framework or theoretical approach: Motivational interviewing. Control group: 860 patients: Usual care. Frequency: Four routine visits. Intensity: Not specified	Group education (10‐15 participants)	12	IG: 385, CG: 319	Health promoter, physician, nurse	(2) Significant difference within IG in SBP (*P* = .04) and DBP (*P* = .002). No significant difference between groups in ICER (cost‐effectiveness) = 1862 $/QALY gained
Muchiri et al, 2016^20^	South Africa (Moretele), Rural	N = 82; n = 71	58.80 (7.70)	T2D	Intervention group: 41 patients: Nutrition education programme and usual care. Frequency: Weekly (8 wk) and monthly (4 mo) and two bi‐monthly. Intensity: 2‐2.5 h (*T* = 26.5 h) Topics: Diabetes mellitus (definition and management), dietary guidelines (healthy eating, mixed meals, portions and meal frequency, healthy cooking with diabetes) and vegetable gardening (improve vegetable and fruit availability, demonstration of sowing/transplantation of vegetables). Supports: Pamphlet and wall/fridge poster. Framework or theoretical approach: Social cognitive theory, the health belief model and the knowledge attitude behaviour model. Control group: 41 patients: Usual care. Frequency: Consultation visit. Intensity: Not specified	Group education (6‐10 participants)	12	IG: 3, CG: 3	Dietician, nurse	(1) No significant difference between groups in HbA1c (*P* = .16). (2) No significant difference between groups in SBP (*P* = .89), DBP (*P* = .28), BMI (*P* = .18), total cholesterol (*P* = .37) and dietary outcomes (*P* > .05). Significant difference between groups in starchy foods (*P* = .01).
Muchiri et al, 2016^26^	South Africa (Moretele), Rural	N = 82; n = 71	58.80 (7.70)	T2D	Intervention group: 41 patients: Nutrition education programme and usual care. Frequency: Weekly (8 wk) and monthly (4 mo) and two bi‐monthly. Intensity: 2‐2.5 h (*T* = 26.5 h) Topics: Diabetes mellitus (definition and management), dietary guidelines (healthy eating, mixed meals, portions and meal frequency, healthy cooking with diabetes) and vegetable gardening (improve vegetable and fruit availability, demonstration of sowing/transplantation of vegetables). Supports: Pamphlet and wall/fridge poster. Framework or theoretical approach: Social cognitive theory, the health belief model and the knowledge attitude behaviour model. Control group: 41 patients: Usual care. Frequency: Consultation visit. Intensity: Not specified	Group education (6‐10 participants)	12	IG: 3, CG: 3	Dietician, nurse	(1) Significant difference between groups in diabetes knowledge scores at 6 mo—baseline (*P* = .033), and at 12 mo—baseline (*P* < .001). Significant difference within IG in patient autonomy (*P* = .028)
Nwamaka Onyechi et al, 2016^37^	Nigeria (Anambra State), Urban	N = 80; n = 55	52.79 (21.89)	T2D	Intervention group: 40 patients: Cognitive behavioural coaching programme. Frequency: One session twice per week Intensity: 50 min. Topics: Enhancing participants' motivation to change; goal setting; monitoring progress; dietary management; disputing unrealistic beliefs; and relapse prevention. Supports: None. Framework or theoretical approach: Rational‐emotive and cognitive behavioural therapy approach. Control group: 40 patients: Conventional counselling. Frequency: Twice per week Intensity: 50 min	Individual education	6	0	Nurse	(1) Significant difference between groups in depressive symptoms (DIDSOC, IG) (*P* < .000)
Ojieabu et al, 2017^38^	Nigeria (Sagamu), Urban	N = 150; n = 93	Not provided	T2D	Intervention group: 75 patients: Pharmacist's educational and counselling. Frequency: Once session a month for 4 mo. Intensity: Not specify. Topics: Diabetes and hypertension, their complications, risks, preventive measures and management; need for medication and treatment adherence such as clinic visits and lifestyle modifications including diet and exercise. Supports: Phone calls. Framework or theoretical approach: Pharmacist's educational and counselling approach. Control group: 75 patients: Deprived of the pharmacist‐led education and counselling sessions throughout the period of the study. Frequency: Once a month. Intensity: 10‐15 min	Individual education	4	0	Pharmacist	(1) Significant difference within IG in FBS (*P* < .001). (2) Significant difference within IG in SBP (*P* < .001) and DBP (0.002). No significant difference between groups in BMI (*P* > .05). Significant difference between groups in medication adherence (*P* = .001), diet (*P* < .001), exercise (*P* < .001) and hospital admissions (*P* = .001)
Total	Nigeria (n = 2), South Africa (n = 2), Ethiopia (n = 2), Rwanda (n = 1), Mali (n = 1) and Kenya (n = 1)	N = 2,743; n = 1,838	53.51 ± 4.70	/	/	Group education: n = 5 and individual education: n = 4	From 3 to 12 mo	891	Nurse (5), physician (4), pharmacist (2), dietician (2), psychologist (1), peer (1), health promoter (1)	/

#### 2.2.3 Analysis

**FIGURE 2:**
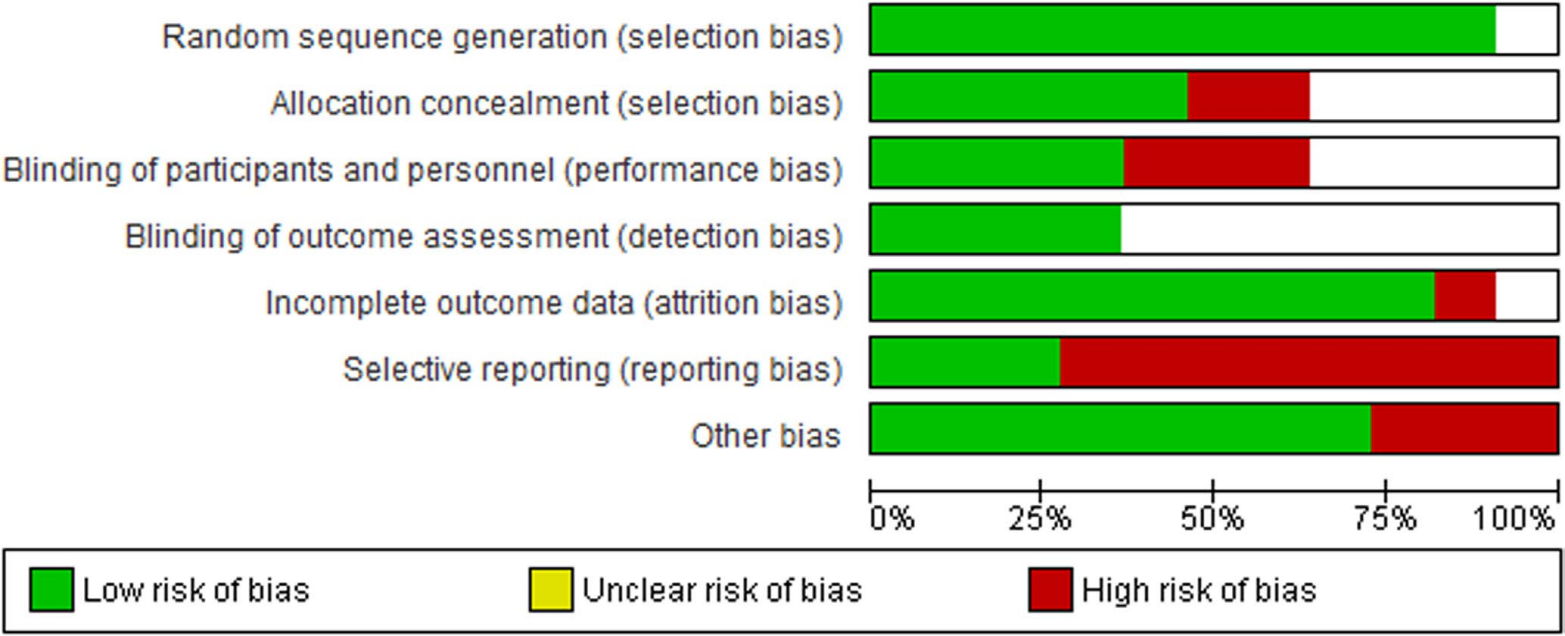
‘Risk of bias’ graph: review authors' judgements about each risk of bias item presented as percentages across all included studies

**FIGURE 3:**
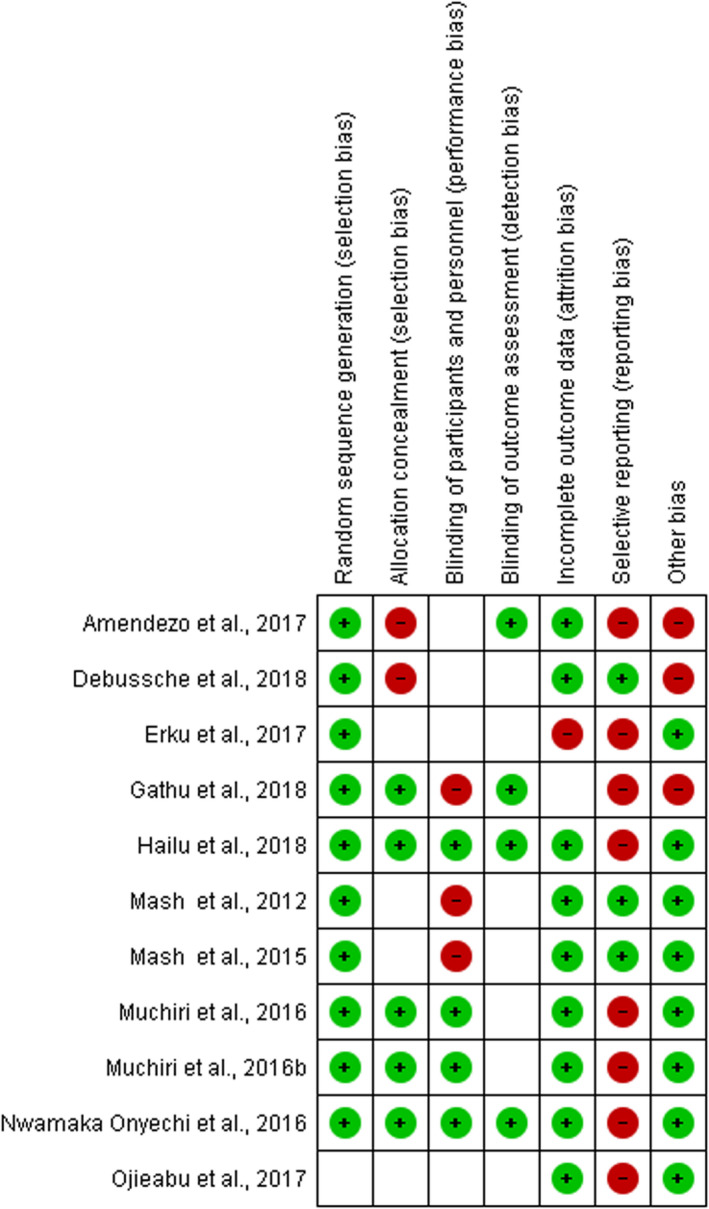
‘Risk of bias’ summary: review authors' judgements about each risk of bias item for each included study

Two authors (AM and CO) independently evaluated the risk of bias in each selected study according to the recommendation of the International Cochrane Collaboration for Systematic Reviews of Interventions.^32^ This involved a description and a judgement for random sequence generation, allocation concealment, blinding, incomplete outcome data, and selective outcome reporting and other potential sources of bias. The criteria for judgement were ‘low risk’, ‘high risk’ or ‘unclear risk’ and presented as percentages across included studies (Figure 2); they were also assessed individually (Figure 3).^32^ Any disagreements between authors were resolved by consensus, or with consultation of the senior author (BKD).

A narrative description of population and study characteristics of selected studies were carried. Review Manager version 5.3 was used to perform statistical analysis.^32^ A random‐effects approach was used for all analyses because it was unlikely that the underlying data represented the true effect due to differences in the populations and interventions in the different studies. For continuous outcomes when the same measurement scale was used (eg HbA1c, BP, BMI), the mean difference was calculated. Results were described narratively for continuous outcomes with different measurement scales such as self‐efficacy, when treatment effects such as standardized mean differences (SMDs) were not quantifiable due to insufficient data to allow formal meta‐analyses.

Heterogeneity was identified by visual inspection of the forest plots and by using the chi‐square test (significance level of 0.1) and *I* ^2^ statistic (0%‐40%: might not be important; 30%‐60%: may represent moderate heterogeneity; 50%‐90%: may represent substantial heterogeneity; and 75%‐100%: considerable heterogeneity).^33^, ^34^ When heterogeneity was found (*I* ^2^ ≥ 50% or *P* < .1), we examined individual study and subgroup characteristics to determine its potential sources. We performed subgroup analysis as a hypothesis‐generating exercise. There were enough data to perform subgroup analyses on the duration and the type of intervention.

### 2.3 Sensitivity analysis

We performed a sensitivity analysis excluding the studies that reported high losses to follow‐up of all participants at the end of the intervention, the studies with nonsignificant results and the community‐based intervention. The robustness of the results was tested by repeating the analysis using fixed‐effects model and random‐effects model.

## 3 RESULTS

### 3.1 Results of the search

The search yielded 1495 publications and 767 after deduplication. A screening based on title and abstract excluded 690 publications, 77 full publications that were assessed for eligibility, and 11 publications from nine studies were reviewed (Figure 1).

### 3.2 Characteristics of included studies

Table 1 summarizes the main characteristics of the 11 publications. In some cases, there were more than one publication from the same study,^20^, ^26^, ^35^, ^36^ leading a total of nine unique studies. These studies yielded a total of 2743 participants, 67% being female (n = 1838). The sample size varied from 80 participants^37^ to 1570 participants.^36^ Ages ranged from 18 to 80 years (mean = 53.51 ± 4.70 years). Six countries were represented in included studies: Nigeria (n = 2), South Africa (n = 2), Ethiopia (n = 2), Rwanda (n = 1), Mali (n = 1) and Kenya (n = 1). All studies were published between 2012 and 2018; they took place in urban areas (n = 7), rural areas (n = 1) or mixed areas (n = 1).

### 3.3 Interventions

#### 3.3.1 Duration, intensity, frequency, types

The duration of interventions ranged from 3 to 6 months (n = 5) to 1 year (n = 4). Fully 32.48% (n = 891) of patients were lost to follow‐up at the end of the intervention, mainly from one study.^35^

The intensity of interventions was very similar across the different studies. The duration of education session varied from 45‐60 minutes (n = 4) to 1‐2.5 hours (n = 4) and was unspecified in one study (Table 1).^38^

The frequency of the interventions varied widely from one study to another. They were classified as not frequent (n = 3) with one course every 3 months^35^, ^39^, ^40^ and relatively frequent (n = 4) with 1^24^, ^38^; 1.5^41^ and 6^42^ monthly education and follow‐up sessions. The most frequent interventions (n = 2) were the ones with weekly education sessions for 8 weeks and monthly follow‐up sessions^26^ and two education sessions a week and follow‐up sessions (Table 1).^37^

Eight interventions were clinic‐based, and one was community‐based.^39^ Interventions were diabetes self‐management education (n = 3),^35^, ^41^, ^42^ pharmacist‐led intervention (n = 2),^38^, ^40^ lifestyle education programmes (n = 2),^24^, ^26^ cognitive behavioural coaching^37^ and peer‐led intervention.^39^

#### 3.3.2 Strategy

Two strategies were adopted: group education (n = 5) and individual education (n = 4).The healthcare professional responsible for this education varied by the nature of the intervention (nurse, physician, pharmacist, dietician, psychologist, peer and health promoter). One intervention^24^ had four types of healthcare professionals (physician, nurse, dietician and psychologist), while several other interventions had only one type of healthcare professional responsible for patient education (Table 1).^37^, ^38^, ^39^, ^41^, ^42^

#### 3.3.3 Theoretical framework

All included studies had a theoretical underpinning for patient empowerment. Key elements of these interventions were mainly derived from patient‐centred approach (n = 3),^24^, ^40^, ^41^ self‐management approach (n = 2),^39^, ^42^ health behaviour models (n = 2),^26^, ^37^ motivational interviewing^35^ and pharmacist's educational and counselling approach (Table 1).^38^

#### 3.3.4 Topic and educational support

All the selected studies included education on diabetes and related factors, plus self‐management of the diseases. Additionally, the interventions provided patients with educational support in terms of material (pamphlets, booklets, etc), immaterial (phone call, text message, etc) and financial compensation (free charge of phone counselling, FBS test, etc); these supports were not provided in one intervention (Table 1).^37^

#### 3.3.5 Outcomes

##### Primary outcomes

HbA1c and FBS as indicators to measure the blood sugar level were mentioned in six studies^24^, ^26^, ^35^, ^39^, ^41^, ^42^ and one study,^38^ respectively; the two other studies measured none of these parameters (Table 1). The effects of interventions on HbA1c were mixed. The studies reported results that favoured the intervention groups, with a statistically significant difference in the improvement of HbA1c (n = 2),^24^, ^39^ results that favoured the intervention but were statistically insignificant (n = 2)^26^, ^42^ and no significant difference between control and intervention groups (n = 2).^35^, ^41^ For FBS, the study reported a significant difference within the intervention group only.^38^

**FIGURE 4:**
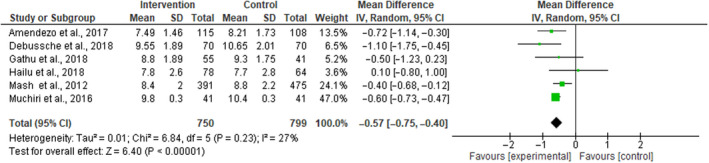
Forest plot of RCTs investigating the effectiveness of patient empowerment interventions on HbA1c

Six studies with 1549 participants contained enough data to be included in a meta‐analysis^24^, ^26^, ^35^, ^39^, ^41^, ^42^ as shown in Figure 4. The pooled results indicate that there is a small, statistically significant difference in the outcomes between intervention and control groups (MD) −0.57 [95% CI: −0.75, −0.40] (*P* < .00001), without important heterogeneity in the effects of the intervention (*I* ^2^ = 27%; Figure 4). A sensitivity analysis excluding the heterogeneous study^35^ reported high losses to follow‐up of all participants at the end of the intervention and dropped the heterogeneity to 20% with higher overall effect size for HbA1c of −0.62 [95% CI: −0.83, −0.42] (*P* < .0001; Figure 4′ Appendix S2). Then, excluding community‐based intervention,^39^ a sensitivity analysis dropped the heterogeneity to 0% with lower overall effect size of −0.59 [95% CI: −0.72, −0.47] (*P* < .00001; Figure 4″ Appendix S3).

Four studies were identified with different measures of self‐management in disease control. Two studies reported diabetes knowledge score, one with a significant difference between control and intervention groups^20^ and the other one without.^39^ One study^35^ reported self‐management in disease control in terms of psychological factor scores (self‐efficacy, internal locus of control, external locus of control, chance locus of control) with no significant difference between control and intervention groups. One study^20^ also reported a significant difference in patient autonomy in their disease management between groups. Depressive symptom scores were used in one study^37^ to measure the self‐management in disease control with a significant difference between control and intervention groups.

##### Secondary outcomes

Seven studies reported blood pressures^24^, ^26^, ^35^, ^38^, ^39^, ^41^, ^42^: three reported a significant difference between groups in the control of systolic blood pressure (SBP) and diastolic blood pressure (DBP)^24^, ^35^, ^42^; one reported a significant difference within the intervention group for SBP and DBP^38^; one found a difference for groups with SBP but not in DBP^39^; and two reported no significant difference between groups for SBP and DBP.^26^, ^41^

Seven studies with SBP and DBP contained enough data to be included in a meta‐analysis of 1699 participants as shown in Figures S4 and S7, respectively. The pooled results for SBP indicated that there is a statistically significant difference in outcomes of mean difference (MD) −5.13 [95% CI: −9.42, −0.84] (*P* = .02) with substantial heterogeneity in the effects of the interventions (*I* ^2^ = 90%; Figure S5, Appendix S4). The pooled results for DBP indicated that there is a statistically significant difference in the outcomes of mean difference (MD) −4.28 [95% CI: −7.18, −1.37] (*P* = .004) with substantial heterogeneity in the effects of the interventions (*I* ^2^ = 91%; Figure S6, Appendix S7). A sensitivity analysis, excluding the heterogeneous studies,^26^, ^41^ reported no significant difference between groups for BP, followed by the study that reported high losses to follow‐up of all participants at the end of the intervention,^35^ respectively, dropped the heterogeneity for SBP to 75% with higher overall effect size of −7.29 [95% CI: −11.34, −3.23] (*P* = .0004; Figure S5′, Appendix S5) and to 65% with higher overall effect size of −8.58 [95% CI: −12.78, −4.38] (*P* < .0001; Figure S5″, Appendix S6). Also, excluding nonsignificant studies^26^, ^41^ and the study that reported high losses to follow‐up of all participants,^35^ respectively, a sensitivity analysis dropped the heterogeneity for DBP to 66% with higher overall effect size of −5.41 [95% CI: −7.71, −3.11] (*P* < .00001; Figure S6′, Appendix S8) and to 28% with higher overall effect size −6.40 [95% CI: −8.43, −4.37] (*P* < .00001; Figure S6″, Appendix S9).

For lipid profile parameters, two studies measured the total cholesterol with no significant difference between groups.^26^, ^35^

For physical parameters, four studies measured BMI^26^, ^38^, ^39^, ^41^ and only one of them reported a statistical significance.^39^ The four studies contained enough data to be included in a meta‐analysis of 468 participants as shown in Figure S7 (Appendix S10). The pooled results indicate no statistically significant difference in outcomes of mean difference (MD) −0.82 [95% CI: −1.71, 0.08] (*P* = .07) with moderate heterogeneity in the effects of the interventions (*I* ^2^ = 50%; Figure S7, Appendix S10). Two studies reported weight, one with statistical significance,^24^ and the other without.^35^

One study measured the cost‐effectiveness of the intervention in terms of incremental cost‐effectiveness ratio (ICER) and reported 1862 $/quality‐adjusted life‐year (QALY) gained,^35^ but fail to show significant differences between groups.

The medication adherence was measured in three studies with significant differences,^38^, ^40^ except in one study^35^ where there was no significant difference between groups.

One study reported hospital admissions with significant reductions in the intervention group compared to the control group.^40^

Lifestyle was evaluated in three studies in terms of adherence to diet plan with significant differences between control and intervention groups in one study^38^ and no significant difference in two other studies,^26^, ^35^ except for starchy foods servings/day that displayed significant differences between groups.^26^ Physical activity plan was reported in two studies, with significant differences between groups in one.^38^ One study^35^ also evaluated the reduction of the frequency of smoking and found no significant difference between groups; they also used some parameters to evaluate the lifestyle (physical functioning, role functioning, social functioning, mental health, general health, pain) but no significant difference was found between groups.

### 3.4 Subgroup analysis

Studies were divided into short‐term measured outcomes (3‐6 months) and long‐term measured outcomes (12 months). When outcomes at 6 months were combined,^26^, ^41^, ^42^ the heterogeneity dropped (*I* ^2^ = 23%), with the significant overall effect size for HbA1c of −0.54 [95% CI: −0.84 to −0.25] (*P* = .0003; Figure S8, Appendix S11). For studies with outcomes measured at 12 months, the heterogeneity was reduced (*I* ^2^ = 34%), and the overall effect size for HbA1c was higher and statistically significant −0.60 [95% CI: −0.78, −0.42] (*P* < .00001; Figure S8, Appendix S11). Lifestyle interventions combined in meta‐analysis seem to be more effective (−0.61 [95% CI: −0.73, −0.49] (*P* < .00001; Figure S9, Appendix S12), *I* ^2^ = 0%) than diabetes self‐management education (DSME; −0.37 [95% CI: −0.62, −0.12] (*P* = .004; Figure S9, Appendix S12), *I* ^2^ = 0%).

### 3.5 Risk of bias in included studies

All included studies were RCTs: most of those in ‘selective reporting’ were at high risk of bias, whereas those in ‘random sequence generation’ and ‘incomplete outcome data’ were at low risk of bias. Most studies did not provide details about the allocation concealment and blinding process (participants and outcomes); thus, it is difficult to make a judgement about how biased some of the studies may be (Figures 2 and 3).

## 4 DISCUSSION

Nine studies with 2743 participants were included in the review. These studies included a wide spectrum of interventions covering clinic‐ and community‐based interventions distributed into DSME, cognitive behavioural coaching, pharmacist, peer‐led and lifestyle interventions.

The result of the glycaemic control indicated small but statistically significant differences in the mean difference of outcomes between intervention and control groups. The subgroup analysis showed that long‐term interventions seem to be more effective than short‐term interventions. Indeed, since diabetes is a chronic NCD and patients are likely to carry it for the rest of their lives, their experiences become rich sources of knowledge to use in developing long‐term interventions.^14^ Recognized as both an outcome by itself and as an intermediate step to long‐term health status, the PE has gained prominence in the healthcare system. This has contributed to the movement away from paternalism towards partnership of care model, building on the recognition of experiential knowledge gained from living with the long‐term disease, which is complementary to scientific knowledge of health professionals.^14^ As an actor of care, the patient may use his experiential knowledge to participate in the care decision‐making process, develop competency, self‐manage his condition and contribute to continuous improvement in the quality of healthcare delivery. It has been shown that the longer the duration of the intervention, the more likely a positive impact of the intervention especially for chronic conditions, because participants have the time to become empower and change their behaviour for producing the expected effects.^43^ Unlike the systematic review of Minet et al^44^ that reported that short‐term self‐management interventions with small groups of participants were likely to be more effective in terms of diabetic control, this review showed that long‐term interventions are the most effective in the context of SSA. Similarly, the lifestyle interventions combined in meta‐analysis seem to be more effective than DSME. This can be explained by the fact that diabetes is a lifestyle‐related disease, so patient empowerment interventions that mainly focus on lifestyle change are more likely to control the disease.^45^

Patient education based on an empowerment approach has previously shown positive effects on the self‐management for the control of the diseases.^29^ In this review, four studies were identified with different measures of self‐management in diabetes control using different parameters and different scales, so it was not possible to do a meta‐analysis. None of these studies simultaneously evaluated the three concepts of patient empowerment approach as defined by Antonovsky.^17^ Only two out of four studies evaluated self‐efficacy and reported a statistically significant difference in outcomes between groups in favour of the intervention group. This may be explained by the frequency and the duration of the patient education, one to twice a week from 50 minutes to 2.5 hours each.^20^, ^37^ More interactions with long duration each with the healthcare professionals improve patient knowledge about the disease (intelligibility) and help better participate in the process of decision‐making as well as improve his self‐efficiency.^46^ A long‐term intervention with a much‐reduced frequency (one session every 3 months) even if the duration of each session is long (1‐2 hours)^35^, ^39^ may not be long enough for the patient to develop the self‐management ability that will help him control the disease. Empowering patient will likely improve his life quality or positively change his health behaviour such as physical activity, diet, smoking and alcohol.^17^ Unfortunately, the two studies that showed a significant improvement in self‐management in this review did not evaluate their impact on behavioural change.^20^, ^37^ Nonetheless, the nonsignificant improvement in patient self‐management had no positive effects on behavioural change (such as diet, physical activity, smoking, medication adherence, quality‐of‐life score) and no significant change in glycaemic control.^35^

Only four of the nine studies evaluated the self‐management of the disease by patients after the intervention and only one study indicated the behavioural change that followed; this makes it difficult to know whether the observed effect actually derived from the intervention.

For the secondary outcomes, two studies with nonsignificant difference in BP after the intervention appeared to be the ones with low frequencies although the duration of education was long; the topic was well related to the self‐management of diabetes with substantial support.^26^, ^41^ The other secondary outcomes were evaluated narratively; they mainly had a mixed effect which makes it difficult to conclude on the effectiveness of the interventions.

Components of patient empowerment intervention have been shown to be particularly effective when delivered by a multidisciplinary team.^47^, ^48^ As such, intervention with more than three types of healthcare professionals, especially with a psychologist, who is important for the ‘meaningfulness’ component of patient empowerment, seemed to be effective in all evaluated variables. Indeed, Antonovsky defined the ‘meaningfulness’ as the motivator that guides the other components of patient empowerment (intelligibility and management); intervention that includes this component in terms of psychosocial support will have a greater impact in the management of the diseases. But in this review, we cannot fully ascertain whether multidisciplinary settings led to the effectiveness of patient empowerment intervention since some included studies consisted of multidisciplinary teams which did not significantly impact the outcomes.^26^, ^35^

All the frameworks identified here were useful to explain how patient behaviours changed to become empowered and responsible of his own health care. This was particularly well with the case of the healthcare patient‐centred approach,^16^ which considers the characteristics, values and experiences of patients. However, the motivational interviewing framework^36^ appears to have more appeal than others because it is considered as a motivational component, which serves as a driving force to combatting diseases and recover lost health.^17^ Meaningfulness, which is central to the three components of the SOC, is also represented by this framework characterized by a collaborative approach that evokes ideas and solutions from the patients, is based on their experiential knowledge, and respects their choices and sense of control while attempting to empathically understand their perspective.^36^

There are several strengths of this review. First and to our knowledge, this review is the first comprehensive review of evidence on patient empowerment interventions for diabetes patients in SSA. Second, we use a structured procedure of data collection according to PRISMA,^30^ and there were no limitations established for date of publication of articles. Third, all the studies were RCTs.

This review also has limitations. First, the search strategy used did not allow us to integrate all the articles related to the topic, and other databases may have been missed, for instance if they were not in French or English. Second, our findings must be situated in the context of the quality of included studies. Some of the included RCTs did not provide details about the allocation concealment, blinding process (participants and outcomes) and selective reporting. Hence, it is difficult to make a judgement about how biased some of the studies may be. Moreover, the small numbers of studies per outcome limited the interpretation of efficacy for the self‐management of interventions investigated. Third, although we searched grey literature, we did not locate any unpublished RCTs that fulfilled the inclusion criteria.

Overall, we believe that the findings from this review are of importance to clinicians, researchers, patients and policymakers directly or indirectly involved in the prevention and control of type 2 diabetes in sub‐Saharan African countries as well as similar resource‐limited settings elsewhere in the world.

## 5 SUMMARY

This review supports the findings that interventions based on patient empowerment may improve HbA1c and BP in patients with diabetes. The long‐term or the lifestyle interventions appear to be the most effective in terms of improving glycaemic control. It was not possible to determine the effectiveness of intervention in all selected outcomes; those classified as most frequent, that utilized support for patient education, and multidisciplinary teams were associated with improved outcomes. However, more evidence from high quality of studies on most interventions with large sample sizes is required to support future patient empowerment programmes. Existing interventions are poorly implemented in the context of SSA, and there is a need to contextualize and standardize their implementation, by using the same definition of patient empowerment and by using the same indicators to evaluate the effects of the intervention.

## CONFLICT OF INTEREST

Nothing to declare.

## AUTHORS' CONTRIBUTION

AM, BKD and MH designed the review and the searches. AM and CO‐O selected relevant studies and performed data extraction. AM analysed the data and wrote the first draft of this review. BKD as senior author participated with MH in writing and editing the manuscript. All authors approved the final version of the manuscript.

## Supporting information

Appendix S1

Appendix S2‐S12

## Data Availability

The data that support the findings of this study are available from the corresponding author, (AM), upon reasonable request.
