# Supplementary material for: Effectiveness of interventions based on patient empowerment in the control of type 2 diabetes in sub‐Saharan Africa: A review of randomized controlled trials
Source: Endocrinol Diabetes Metab. 2020 Aug 25;4(1):e00174. doi: 10.1002/edm2.174 (PMC7831206; doi:10.1002/edm2.174)
Supplement: Supplementary file 1 — Appendix S1 [file EDM2-4-e00174-s001.docx]

**Appendix S1- Definitions**

***Diagnostic criteria.*** To be included in this study, the diagnosis of diabetes mellitus should have been established using the valid standard criteria (28). Ideally, diagnostic criteria should have been described and where necessary, authors’ definition of diabetes mellitus were used.

***Intelligibility.*** These included patient education interventions, either individually or in groups, by health professionals (e.g. physician, nurse) or not (e.g. social worker, peer), based on the development of personal skills/abilities for the control of the disease and the improvement of knowledge about the diabetes and associated factors, complications and treatment options. Based on the theories for behavior change (10-13), only interventions where patients are followed for at least three months, minimum time to develop the personal aptitudes for controlling the disease, were retained.

***Manageability.*** The interventions could be pharmacological or non-pharmacological. The pharmacological interventions are those that target detecting and treatment, surveillance and control of the disease (e.g. Monitoring blood glucose), access to services and patient adherence to therapeutic plan. Non-pharmacologic interventions mainly focus on changing lifestyles related to diet, physical activity, alcohol use and smoking. All the intervention looking for the action of disease management as taking medication, self-monitoring of blood glucose, insulin titration, measurement of foods intake, frequent exercise, etc.

***Meaningfulness.*** Meaningfulness has an emotional sense like a way of looking at life as worth living, by providing the motivational force (13). This integrated all interventions that provide a psychosocial adjustment to daily life, managing anxiety, depression and stress, providing good social support for patients.

***Resources.*** Koelen and Lindström (15) have identified four examples of internal resources: health locus of control, learned helplessness, self-efficacy and outcome expectations. The externals resources are the ones related to health care actors and health care system as the practices of health care professionals, institutional culture and policies.
