# Supplementary material for: Effectiveness of interventions based on patient empowerment in the control of type 2 diabetes in sub‐Saharan Africa: A review of randomized controlled trials
Source: Endocrinol Diabetes Metab. 2020 Aug 25;4(1):e00174. doi: 10.1002/edm2.174 (PMC7831206; doi:10.1002/edm2.174)

**Appendix S2 toS12 - Figures**

**Figure 4’.** **Appendix S2:** Forest plot of RCTs investigating the effectiveness of patient empowerment interventions on HbA1c (With the removal of the heterogeneous study (33))


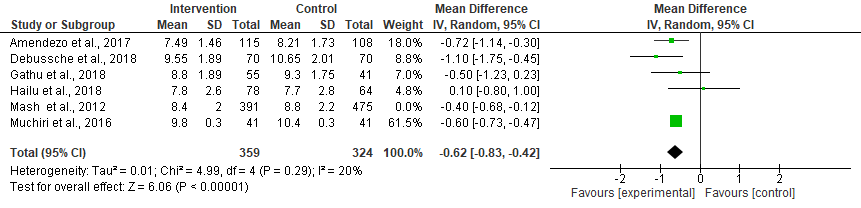


**Figure 4’’. Appendix S3:** Forest plot of RCTs investigating the effectiveness of patient empowerment interventions on HbA1c (With the removal of the heterogeneous studies (33, 37))


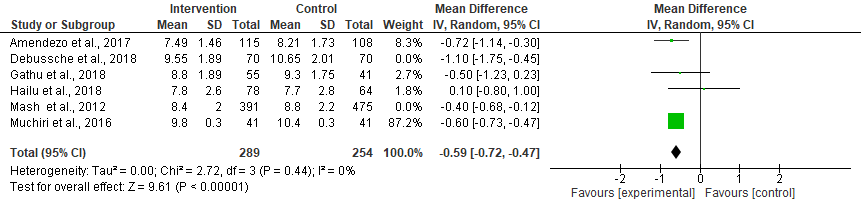


**Figure 5. Appendix S4:** Forest plot of RCTs investigating the effectiveness of patient empowerment interventions on SBP


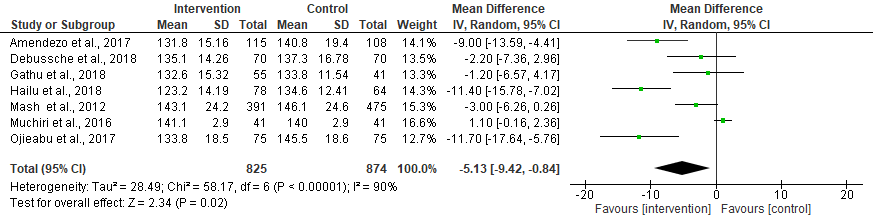


**Figure 5’. Appendix S5:** Forest plot of RCTs investigating the effectiveness of patient empowerment interventions on SBP (With the removal of the heterogeneous studies (25, 39))


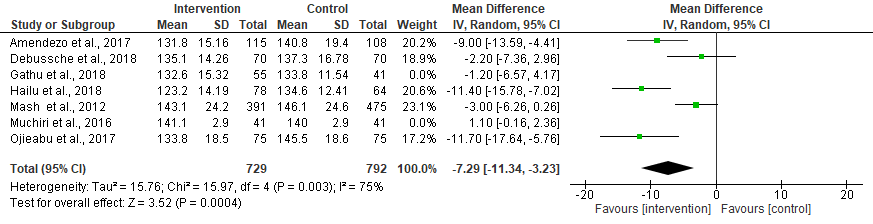
**Figure 5’’. Appendix S6:** Forest plot of RCTs investigating the effectiveness of patient empowerment interventions on SBP (With the removal of the heterogeneous studies (25, 33, 39))**
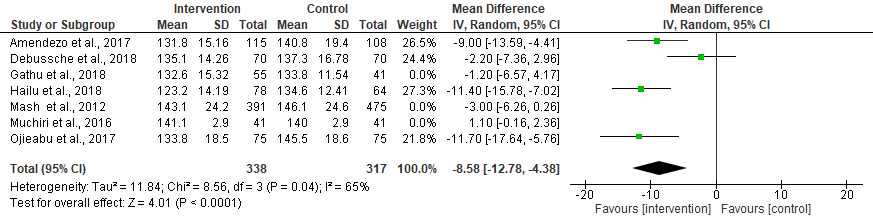
**

**Figure 6. Appendix S7:** Forest plot of RCTs investigating the effectiveness of patient empowerment interventions on DBP


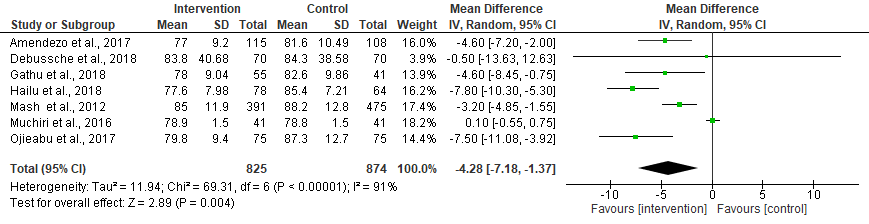


**Figure 6’. Appendix S8:** Forest plot of RCTs investigating the effectiveness of patient empowerment interventions on DBP (With the removal of the heterogeneous studies (25, 39))


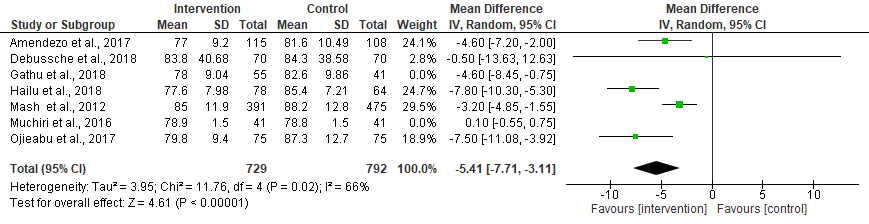


**Figure 6’’. Appendix S9:** Forest plot of RCTs investigating the effectiveness of patient empowerment interventions on DBP (With the removal of the heterogeneous studies (25, 33, 39))


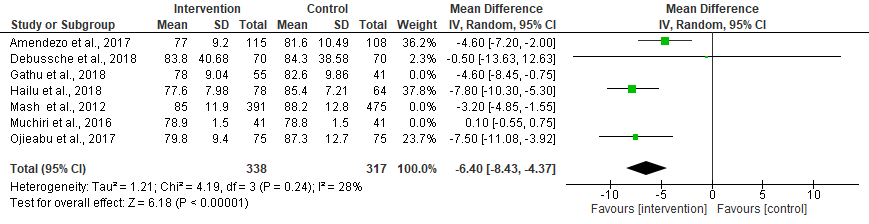


**Figure 7. Appendix S10:** Forest plot of RCTs investigating the effectiveness of patient empowerment interventions on BMI


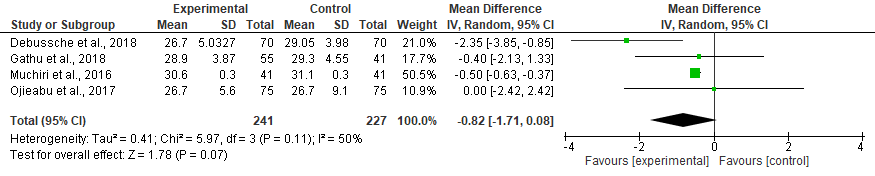


**Figure 8. Appendix S11:** Forest plot of sub-group analyzing: HbA1c at less than 6 months and at 12 months


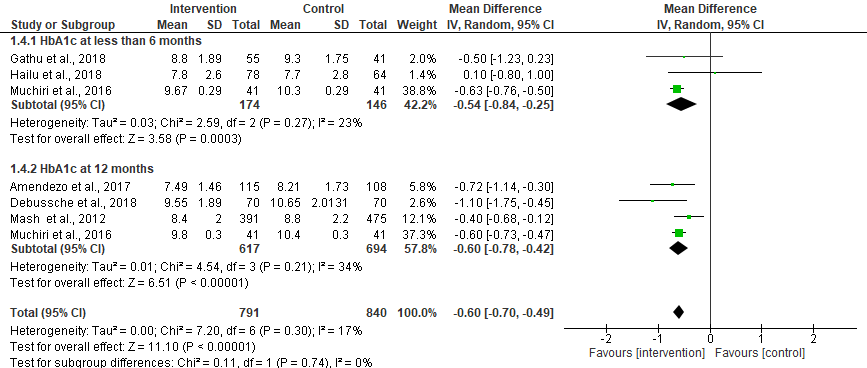


**Figure 9. Appendix S12:** Forest plot of sub-group analyzing: DSME and Lifestyle intervention


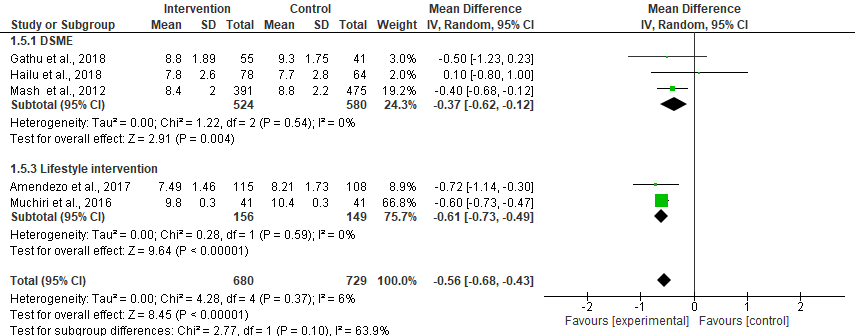

Supplement: Supplementary file 2 — Appendix S2‐S12 [file EDM2-4-e00174-s002.docx]
